# Engineering *Halomonas* species TD01 for enhanced polyhydroxyalkanoates synthesis via CRISPRi

**DOI:** 10.1186/s12934-017-0655-3

**Published:** 2017-04-06

**Authors:** Wei Tao, Li Lv, Guo-Qiang Chen

**Affiliations:** 1 School of Life Sciences, Tsinghua University, Beijing, 100084 China; 2 Center for Synthetic and Systems Biology, Tsinghua University, Beijing, 100084 China; 3 Tsinghua-Peking Center for Life Sciences, Tsinghua University, Beijing, 100084 China; 4 Center for Nano- and Micro-Mechanics, Tsinghua University, Beijing, 100084 China; 5 MOE Key Lab of Industrial Biocatalysis, Department Chemical Engineering, Tsinghua University, Beijing, 100084 China

**Keywords:** CRISPRi, PHBV, PHB, Synthetic biology, *ftsZ*, *gltA*, *prpC*

## Abstract

**Background:**

Clustered regularly interspaced short palindromic repeats interference (CRISPRi) has provided an efficient approach for targeted gene inhibition. A non-model microorganism *Halomonas* species TD01 has been developed as a promising industrial producer of polyhydroxyalkanoates (PHA), a family of biodegradable polyesters accumulated by bacteria as a carbon and energy reserve compound. A controllable gene repression system, such as CRISPRi, is needed for *Halomonas* sp. TD01 to regulate its gene expression levels.

**Results:**

For the first time CRISPRi was successfully used in *Halomonas* sp. TD01 to repress expression of *ftsZ* gene encoding bacterial fission ring formation protein, leading to an elongated cell morphology with typical filamentous shape similar to phenomenon observed with *Escherichia coli*. CRISPRi was employed to regulate expressions of *prpC* gene encoding 2-methylcitrate synthase for regulating 3-hydroxyvalerate monomer ratio in PHBV copolymers of 3-hydroxybutyrate (HB) and 3-hydroxyvalerate (HV). Percentages of HV in PHBV copolymers were controllable ranging from less than 1 to 13%. Furthermore, repressions on *gltA* gene encoding citrate synthase channeled more acetyl-CoA from the tricarboxylic acid (TCA) cycle to poly(3-hydroxybutyrate) (PHB) synthesis. The PHB accumulation by *Halomonas* sp. TD01 with its *gltA* gene repressed in various intensities via CRISPRi was increased by approximately 8% compared with the wild type control containing the CRISPRi vector without target.

**Conclusions:**

It has now been confirmed that the CRISPRi system can be applied to *Halomonas* sp. TD01, a promising industrial strain for production of various PHA and chemicals under open and continuous fermentation process conditions. In details, the CRISPRi system was successfully designed in this study to target genes of *ftsZ*, *prpC* and *gltA,* achieving longer cell sizes, channeling more substrates to PHBV and PHB synthesis, respectively. CRISPRi can be expected to use for more metabolic engineering applications in non-model organisms.

**Electronic supplementary material:**

The online version of this article (doi:10.1186/s12934-017-0655-3) contains supplementary material, which is available to authorized users.

## Background

The CRISPRi (clustered regularly interspaced short palindromic repeats interference) system provides an efficient method for targeted gene repression [1]. Deriving from the CRISPR/Cas9 system, the CRISPRi system contains a dCas9 protein co-expressed with a small guide RNA (sgRNA) [1–3]. The Cas9 protein is an RNA-guided DNA endonuclease. In the CRISPR system, the Cas9 protein binds to the sgRNA and form a protein-RNA complex, which will then bind to the targeted DNA sequence. The DNA will be cleaved by the catalytically active Cas9 protein [4]. Mutations in the Cas9 protein result in a catalytically dead dCas9 protein with DNA binding capability [1]. Therefore, the dCas9/sgRNA complex can bind to specific DNA target depending on the designed sequence of sgRNA, block transcriptional elongation, interfere RNA polymerase or transcriptional factor binding [1].

CRISPRi enables convenient and specific gene regulation in microbial metabolic engineering [1]. In our early study, CRISPRi was successfully used in *Escherichia coli* for regulating polyhydroxyalkanoates (PHA) production via simultaneously repressing multiple genes or multiple targets on one gene [5].

PHA are polyesters synthesized by a wide range of bacteria as carbon and energy source reserves [6]. PHA has been developed into various environmentally friendly plastic products, and it has been formed into an application value chain [7–9]. PHA can be classified into short-chain-length (scl) PHA and medium-chain-length (mcl) PHA [10]. Poly(3-hydroxybutyrate) (PHB) and poly(3-hydroxybutyrate-co-3-hydroxyvalerate) (PHBV) are common PHA already produced in large scale [9]. PHA production cost is still too high compared with petrochemical plastics that are not biodegradable [11].

*Halomonas* sp. TD01 is a halophile screened from Aydingol Lake in Xinjiang Province, China [12]. It can be grown under conditions of high salt concentrations and high pH, allowing a continuous and open fermentation without contamination. The genome of *Halomonas* sp. TD01 was sequenced and some genetic manipulation technologies have been developed for DNA manipulation [13, 14]. The absence of controllable repression system for gene expression has slowed down more applications for *Halomonas* sp. TD01.

*FtsZ* is a tubulin-like protein that is of great importance in the cell division process [15, 16]. *FtsZ* assembles to form Z rings in a dynamic state during the cell division process. *FtsZ* inhibition or deletion in *E. coli* leads to cell division repression and results in formation of filamentous cells from bar or spherical shapes [17, 18].

In *Halomonas* sp. TD01, propionic acid is transformed into propionyl-CoA, which can be further catalyzed by 2-methylcitrate synthase to form 2-methylcitrate and then enters the methyl citric acid cycle (MCC cycle) [19]. Propionyl-CoA can also enter the PHBV synthesis pathway to form 3-hydroxyvalerate monomers. 2-Methylcitrate synthase is encoded by *prpC* gene. It was expected that repressions on *prpC* should divert more propionyl-CoA to PHBV synthesis [20, 21].

The tricarboxylic acid (TCA) cycle provides energy and intermediates for synthesis of many important biological compounds [22]. In TCA cycle, acetyl-CoA and oxaloacetate are converted to citrate by citrate synthase encoded by *gltA* gene [22, 23]. Acetyl-CoA is the substrate for PHB synthesis, a high concentration of acetyl-CoA is of great importance to PHB production. Repressions of *gltA* gene should decrease acetyl-CoA consumption by the TCA cycle, therefore, improving substrate conversion to PHB synthesis.

In this study, it was aimed to exploit CRISPRi for enhanced PHA production by engineering *Halomonas* sp. TD01 to achieve regulation of its expression levels of various genes, including *ftsZ*, *prpC* and *gltA.*

## Results

### Feasibility study of the constructed CRISPRi for *Halomonas* sp. TD01

Gene *ftsZ* encoding bacterial fission ring protein was selected as a reporter gene for feasibility study of CRISPRi system for *Halomonas* sp. TD01. During the bacterial cell division process, FtsZ assembly leads to the formation of Z rings in the middle of a cell. FtsZ inhibitors interact with FtsZ in cytokinesis repressing the cell division progression, resulting in formation of filamentous cells [17, 18].

The sgRNAs were designed in the promoter region or near the ATG sequence in the targeted gene, and were right after a NGG sequence, namely, PAM sequence (protospacer adjacent motif sequence) [4]. All the sgRNAs could bind to the non-template DNA strand with sequence specificity. Thus, two sgRNAs were designed near the ATG sequence in *ftsZ* gene (Fig. 2b). CRISPRi inhibition systems pli-dCas9-ftsZ1 and pli-dCas9-ftsZ2 were constructed. The plasmids were then transferred via *E. coli* conjugation into *Halomonas* sp. TD01, forming the recombinants *Halomonas* sp. TD-ftsZ1 and TD-ftsZ2 strains. *Halomonas* sp. TD01 containing the non-target plasmid pli-dCa9-sgRNA, was named *Halomonas* sp. TD-sgRNA strain. Wild type *Halomonas* sp. TD01 and *Halomonas* sp. TD-sgRNA were used as control groups.

Growth curves of the strains were determined to observe whether cell growth was affected by inhibition of *ftsZ* gene (Additional file 1: Figure S1). Compared with the wild type *Halomonas* sp. TD01 and TD-sgRNA control groups, *Halomonas* sp. TD-ftsZ1 and TD-ftsZ2 exhibited long lag growth and lower cell density, implying that *ftsZ* gene inhibition decreased cell growth rate.

All the strains were cultured in the MM medium with 30 g/L glucose at 37 °C and 200 rpm. After 8 h cultivation, 1 mM IPTG was added for inducing the CRISPRi system. Cells were harvested after an overall cultivation of 48 h. Under an environmental scanning electron microscope (ESEM), *Halomonas* sp. TD-ftsZ1 and TD-ftsZ2 harboring the CRISPRi system showed elongated shapes compared with their controls *Halomonas* sp. TD01 and TD-sgRNA (Fig. 3a), demonstrating that the cell division process was effectively repressed via the CRISPRi. Lengths of bacteria in *Halomonas* sp. TD01 and TD-sgRNA control groups were approximately 1 μm, while lengths of bacteria in *Halomonas* sp. TD-ftsZ1 and TD-ftsZ2 groups varied from 20 to 70 μm, showing 20–70 folds increase in their cell lengths (Fig. 3b). The phenotype changes clearly indicated that the CRISPRi system had been successfully developed in *Halomonas* sp. TD01.

### The uses of *Halomonas* CRISPRi system for controlling PHBV monomer ratios

**Fig. 1:**
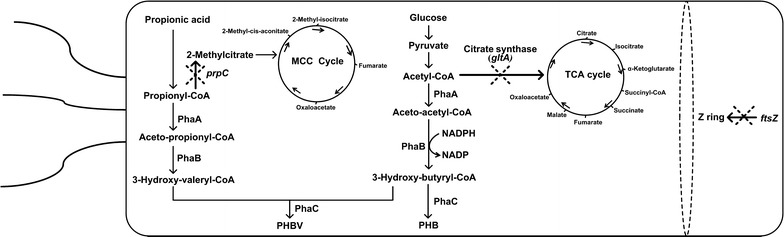
PHBV and PHB metabolic pathways. *Dotted crosses* show the repressed pathways in *Halomonas* sp. TD01 when targeting the indicated genes using the CRISPRi repression system. MMC, methylcitric acid cycle; *prpC*, 2-methylcitrate synthase; *phaA*, β-ketothiolase; *phaB*, NADPH-dependent acetoacetyl-CoA reductase; *phaC*, PHA synthase gene; TCA, tricarboxylic acid cycle; *ftsZ*, filamenting temperature-sensitive mutant Z

*Halomonas* sp. TD01 is able to produce PHBV by adding the substrate propionic acid in the presence of glucose [14]. The PHBV synthesis involves the conversion of propionic acid to propionyl-CoA and subsequent transformation into 3-hydroxyvaleryl-CoA by β-ketothiolase (PhaA) and NADPH-dependent acetoacetyl-CoA reductase (PhaB). PHA synthase (PhaC) polymerizes 3-hydroxyvaleryl-CoA with 3-hydroxybutyryl-CoA to form PHBV (Fig. 1). Propionyl-CoA directly leads to the 3HV monomers in PHBV. In *Halomonas* sp. TD01, 3HV monomer amounts in PHBV was very low due to the rapid conversion of propionyl-CoA to 2-methylcitrate, which is converted to the methylcitric acid cycle (MCC cycle) [14]. In *Halomonas* sp. TD01, 2-methylcitrate synthase is encoded by *prpC* gene. *PrpC* knockout *Halomonas* sp. TD01 showed an accumulation of PHBV with a higher 3HV ratio when in presence of 1 g/L propionic acid. However, in this condition, the cells grew poorly and the cell dry weight (CDW) was very low [14]. Therefore, it is expected that the use of a CRISPRi repression system targeting *prpC* gene in *Halomonas* sp. TD01 could channel more propionic acid, or propionyl-CoA, to 3HV monomer in PHBV synthesis without impairing cell growth (Fig. 1).

As shown in Fig. 4a, seven sgRNAs were designed along the *prpC* gene, mostly near the start site of the coding sequence or within 260 bp from the ATG sequence. CRISPRi plasmids are constructed and named as pli-dCas9-prpC1, pli-dCas9-prpC2, pli-dCas9-prpC3, pli-dCas9-prpC4, pli-dCas9-prpC5, pli-dCas9-prpC6 and pli-dCas9-prpC7, respectively (Additional file 1: Table S1; Fig. 4a). Various sgRNA binding sites led to different repressive effects. Two effective inhibition sites, namely prpC6 and prpC7, were combined together to form pli-dCas9-prpC6prpC7 to verify possible enhanced combinatory inhibition effect. All the plasmids were transferred via *E. coli* conjugation into *Halomonas* sp. TD01, forming strains TD-prpC1, TD-prpC2, TD-prpC3, TD-prpC4, TD-prpC5, TD-prpC6, TD-prpC7 and TD-prpC6prpC7, respectively.

To study the impact of *prpC* gene repression on cell growth, growth curves of the strains were established (Additional file 1: Figure S2). Wild type *Halomonas* sp. TD01 and TD-sgRNA were used as the control groups. The growth trends of the bacteria in all study groups were nearly the same, indicating that the CRISPRi system functioned without affecting cell growth.

All the strains were cultured in the MM medium in the presence of 1 g/L propionic acid and 30 g/L glucose at 37 °C and 200 rpm. After 12 h cultivation, 1 mM IPTG was added to induce the CRISPRi system. All bacteria were harvested after an overall cultivation time of 48 h (Table 1). All the strains grew well with CDW reaching approximately 14 g/L and accumulating approximately 75% PHBV. Meanwhile, *Halomonas* sp. TD01 and TD-sgRNA strains were used as controls, accumulating around 1% 3HV in PHBV copolymer. The percentage of 3HV monomer in PHBV copolymer varied from less than 1% to nearly 13% depending on the *prpC* repression intensity. In the single sgRNA inhibition system, *Halomonas* strains TD-prpC2, TD-prpC3, TD-prpC4 and TD-prpC5 all showed an obvious improvement in 3HV ratio compared to the controls. *Halomonas* strain TD-prpC2 accumulated nearly 5% 3HV in the PHBV, while 3HV ratios in PHBV copolymer accumulated by *Halomonas* strains TD-prpC3 and TD-prpC4 were around 6%. 3HV ratio in the PHBV produced by *Halomonas* strain TD-prpC5 was five times higher than that in the control groups, reaching 8% (Table 1). *Halomonas* strains TD-prpC6 and TD-prpC7 exhibited much higher repression efficiency, with around 12% 3HV in PHBV copolymer. In the two targets inhibition system, *Halomonas* strain TD-prpC6prpC7 produced PHBV copolymer consisting of 12.7% 3HV monomer (Table 1). The combination of prpC6 and prpC7 targets showed a slightly enhanced repression effect compared with that of the individual single sgRNA repression systems.

**Table 1:** Shake flask PHBV production by recombinants *Halomonas* sp. TD01 controlling *prpC* gene expression via CRISPRi

Recombinant TD01 strains	CDW (g/L)	PHBV (wt%)	3HV (mol%)
TD01	13.22 ± 0.20	76.08 ± 2.81	0.83 ± 0.08
TD-sgRNA	13.95 ± 0.71	74.73 ± 2.25	1.29 ± 0.47
TD-prpC1	14.99 ± 0.30	72.14 ± 2.37	1.79 ± 0.02
TD-prpC2	14.43 ± 0.45	75.06 ± 3.20	4.78 ± 0.83
TD-prpC3	14.08 ± 1.26	73.96 ± 7.18	5.72 ± 0.30
TD-prpC4	13.58 ± 0.57	80.14 ± 8.62	6.44 ± 0.43
TD-prpC5	13.59 ± 0.51	80.12 ± 3.27	8.16 ± 0.31
TD-prpC6	14.17 ± 0.23	82.20 ± 3.82	11.94 ± 0.80
TD-prpC7	13.54 ± 0.31	76.74 ± 0.80	12.15 ± 0.31
TD-prpC6prpC7	14.67 ± 0.78	73.78 ± 4.82	12.70 ± 0.27

The recombinants harboring CRISPRi system were cultivated in MM medium containing 30 g/L glucose and 1 g/L propionic acid at 37 °C for 48 h as described in “Methods”. CDW, cell dry weight; PHBV (wt%), the weight percent of PHBV in CDW; TD01, *Halomonas* sp. TD wild type strain; TD-sgRNA, TD01 strain harboring the pli-dCas9-sgRNA plasmid without any DNA target site; TD-prpC1, TD-prpC2, TD-prpC3, TD-prpC4, TD-prpC5, TD-prpC6, TD-prpC7, TD-prpC6prpC7, TD01 strains harboring the pli-dCas9-sgRNA plasmid with different sgRNA targets of gene *prpC*, respectively. All data were the average of three independent studies with standard deviations. Mean ± SE (n = 3)

The mRNA expression levels RT-PCR agreed with the shake flask results (Fig. 4b). Wild type *Halomonas* sp. TD01 and the control TD-sgRNA showed a similar *prpC* mRNA expression level, indicating that the non-target system did not influence the gene expression in the strain *Halomonas* sp. TD-sgRNA. Strains *Halomonas* sp. TD-prpC1 and TD-prpC2 showed a higher mRNA expression level compared with strains TD-prpC3, TD-prpC4 and TD-prpC5, while strains TD-prpC6, TD-prpC7 and TD-prpC6prpC7 revealed a much lower mRNA expression level. This phenomenon again demonstrated the clear feasibility of the CRISPRi system for *Halomonas* sp. TD01. In addition, considering that *prpC* mRNA expression level in *Halomonas* strains TD-prpC6 and TD-prpC7 were already repressed to a very low level, it was hypothesized that further repression could hardly be expected when combining prpC6 and prpC7 inhibition targets. Thus the 3HV ratio in PHBV copolymer produced by *Halomonas* strain TD-prpC6prpC7 was almost the same as that in *Halomonas* strain TD-prpC6 or TD-prpC7.

### The uses of *Halomonas* CRISPRi system for enhanced PHB synthesis

*Halomonas* sp. TD01 is able to produce PHB using glucose as substrate [13]. Acetyl-CoA, as a PHB substrate, is generated from pyruvate after glycolysis and oxidized in the TCA cycle. The citrate synthase catalyzes conversion of acetyl-CoA and oxaloacetate to citrate (Fig. 1). Gene *gltA* encoding citrate synthase, can not be completely repressed in *Halomonas* sp. TD01. However, partial repression on *gltA* should decrease the consumption of acetyl-CoA for TCA cycle and thus save some acetyl-CoA substrate for PHB synthesis (Fig. 1).

Four sgRNAs targeting *gltA* were designed. GltA1 was located in the promoter region, gltA2 sequence started with ATG, while gltA3 was just three base pairs away from gltA2. Finally, gltA4 was designed within 200 bp from the ATG sequence. The respective plasmids were constructed and named as pli-dCas9-gltA1, pli-dCas9-gltA2, pli-dCas9-gltA3 and pli-dCas9-gltA4. They were transferred into *Halomonas* sp. TD01 via *E. coli* conjugation, forming *Halomonas* strains TD-gltA1, TD-gltA2, TD-gltA3 and TD-gltA4.

Growth curves of the strains were established to investigate the effect of *gltA* inhibition on cell growth (Additional file 1: Figure S3). The bacterial growth curves showed that *Halomonas* strains TD-gltA1, TD-gltA2, TD-gltA3 and TD-gltA4 exhibited long lag growth phase in the beginning of the culture compared with the growth curves of *Halomonas* sp. TD01 and TD-sgRNA strains. Also, a similar cell density was reached after 15 h of cultivation, indicating that *gltA* repression prolonged the lag phase in cell growth, yet it did not affect cell density after the overnight cultivation.

All strains were cultured in the MM medium with 30 g/L glucose at 37 °C and 200 rpm. After 12 h cultivation, 1 mM IPTG was added to induce the CRISPRi system. Compared with the control *Halomonas* sp. TD-sgRNA, PHB content in *Halomonas* sp. TD-gltA2 showed a nearly 8% improvement, while strain TD-gltA3 had a 5% increase (Table 2). The mRNA expression levels from RT-PCR demonstrated that the *gltA* was repressed in the recombinant strains harboring the CRISPRi inhibition system targeting *gltA* gene (Additional file 1: Figure S4). Wild type *Halomonas* sp. TD01 and TD-sgRNA showed a similar *gltA* mRNA expression level, yet *Halomonas* strains TD-gltA1, TD-gltA2, TD-gltA3 and TD-gltA4 all showed a decreased *gltA* mRNA expression level. Therefore, partial CRISPRi repression on *gltA* had indeed reduced the consumption of acetyl-CoA thus improved PHB production in *Halomonas* sp. TD-sgRNA. Once again, *gltA* repressions demonstrated that the CRISPRi system was useful for metabolic engineering of *Halomonas* sp. TD01.

**Table 2:** Shake flask PHB production by recombinants *Halomonas* sp. TD01 with controllable *gltA* gene expression via CRISPRi

Recombinant TD01 strains	CDW (g/L)	PHB (wt%)
TD01	10.22 ± 0.25	77.68 ± 3.75
TD-sgRNA	13.28 ± 0.57	63.80 ± 3.08
TD-gltA1	13.68 ± 0.21	66.79 ± 1.59
TD-gltA2	13.53 ± 0.41	71.77 ± 7.16
TD-gltA3	13.18 ± 0.33	69.22 ± 3.43
TD-gltA4	13.10 ± 0.42	66.16 ± 8.11

All strains were cultivated in MM medium containing 30 g/L glucose at 37 °C for 48 h as described in “Methods”. CDW, cell dry weight; PHBV (wt%), the weight percent of PHBV in CDW; TD01, *Halomonas* sp. TD wild type strain; TD-sgRNA, TD01 strain harboring the pli-dCas9-sgRNA plasmid without any DNA target site; TD-gltA1, TD-gltA2, TD-gltA3, TD-gltA4, TD01 strains harboring the pli-dCas9-sgRNA plasmid with different sgRNA targets of gene *gltA*, respectively. All data were the average of three independent studies with standard deviations. Mean ± SE (n = 3)

## Discussion

*Halomonas* sp. TD01 has been demonstrated to be a promising strain for PHA production due to its tolerance to high pH and high salt concentration. Therefore, allowing an open and continuous fermentation process without contamination [13]. This improves competitiveness of *Halomonas* sp. TD01 based PHA production process [24, 25]. However, as a non-model microorganism, *Halomonas* sp. TD01 still requires an inducible gene repression system for better performances.

**Fig. 2:**
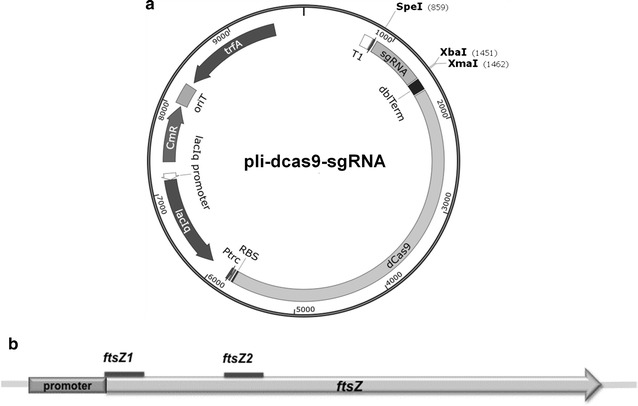
CRISPRi system used for *Halomonas* sp. TD01 (**a**) and relative binding positions of sgRNAs targeting *ftsZ* gene (**b**). Pli-dCas9-sgRNA, plasmid carrying the CRISPRi system; P_trc_, trc promoter; CmR, chloramphenicol resistance gene; oriT, origin of transfer; *ftsZ*, filamenting temperature-sensitive mutant Z. The length of *ftsZ* gene is 1179 bp, while the length of sgRNAs is around 20 bp. The promoter of *ftsZ* gene is 35 bp to 10 bp upstream of *ftsZ* gene. To inhibit *ftsZ* gene expression, *ftsZ1* is designed 4 bp upstream from ATG sequence, from position −26 to −5, after the PAM sequence CGG (from position −29 to −27). *FtsZ2* is designed 89 bp downstream of ATG sequence, from position 90 to 112, after the PAM sequence TGG (from position 87 to 89)

**Fig. 3:**
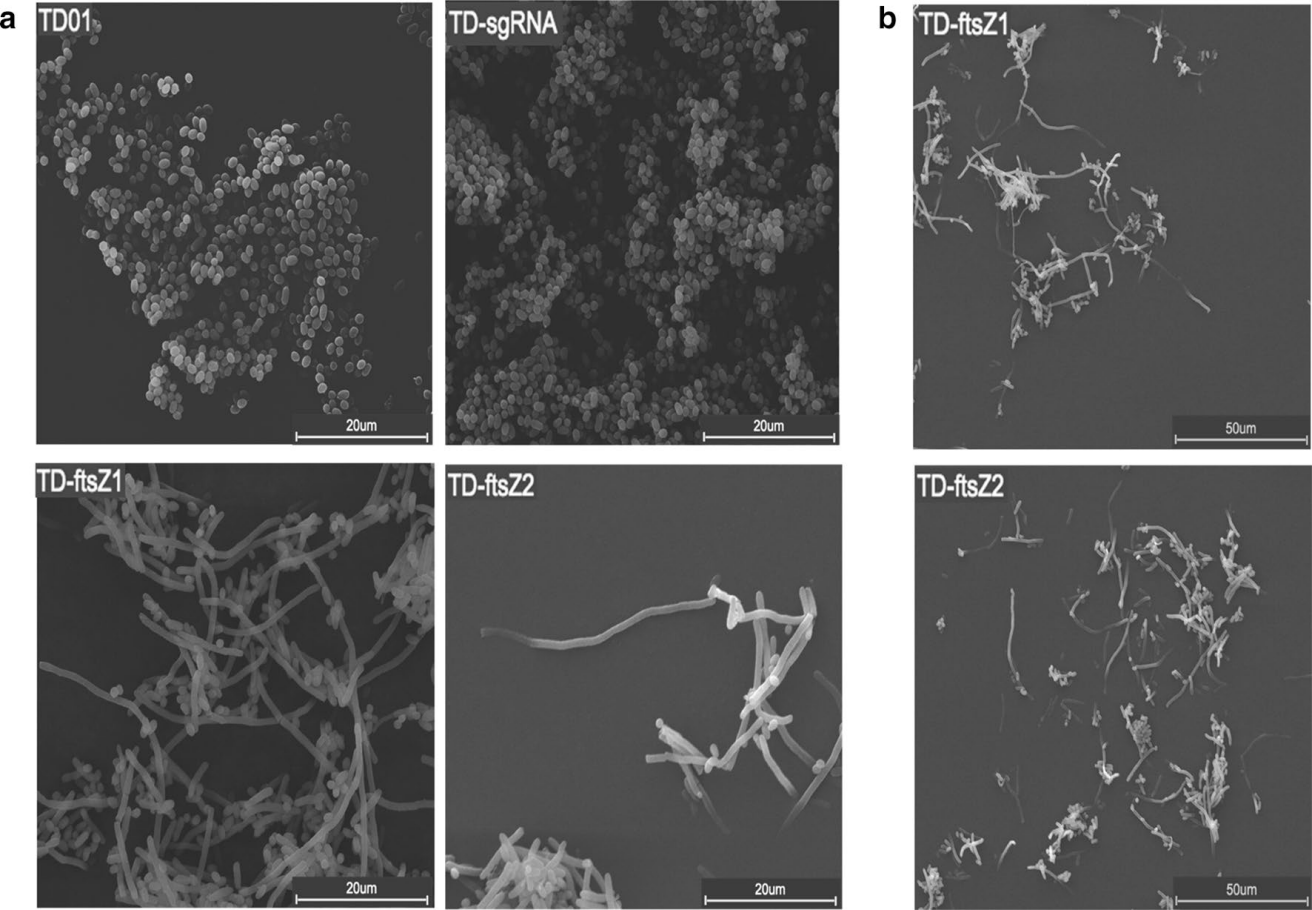
Scanning electron microscopy study on *Halomonas* sp. TD01 with its *ftsZ* gene repressed via CRISPRi under 5000 (**a**) and 2000 (**b**) times magnification. TD01: wild type *Halomonas* sp. TD used as a control group; TD-sgRNA, TD01 strain harboring the pli-dCas9-sgRNA plasmid without any DNA target site; TD-ftsZ1, TD-ftsZ2, TD01 strain harboring the CRISPRi plasmids pli-dCas9-ftsZ1 and pli-dCas9-ftsZ2 that regulated the expression level of fission ring protein *ftsZ* gene, respectively. *Scale bars* are 50 or 20 µm, as indicated

**Fig. 4:**
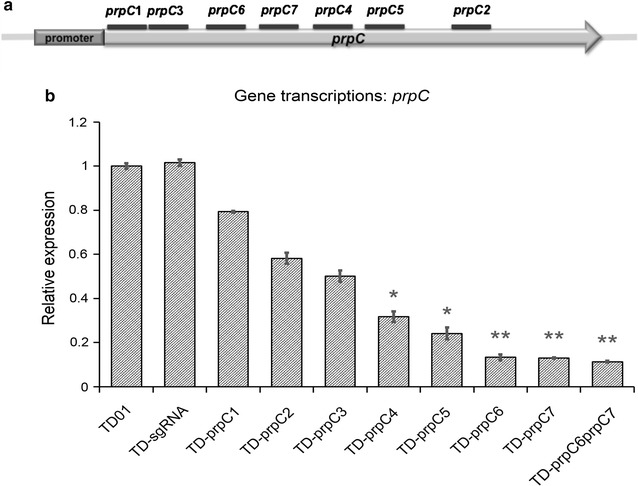
Controllable repression of *prpC* gene transcription in recombinant *Halomonas* sp. TD01. The relative binding positions of sgRNAs targeting *prpC* gene (**a**) and RT-PCR study of *prpC* transcription levels (**b**). All data were the average of three independent studies with standard deviations. Mean ± SE (n = 3). *p < 0.05 and **p < 0.01. TD01, *Halomonas* sp. TD wild type; TD-sgRNA, TD01 strain harboring the pli-dCas9-sgRNA plasmid without any target site; TD-prpC1, TD-prpC2, TD-prpC3, TD-prpC4, TD-prpC5, TD-prpC6, TD-prpC7, TD-prpC6prpC7, TD01 strains harboring the pli-dCas9-sgRNA plasmid with different DNA targets on gene *prpC*

CRISPRi has been used to regulate expression of desired genes without affecting the normal growth of engineered cells [1, 26–28]. In this study, an effective CRISPRi platform for genome editing in *Halomonas* sp. TD01 was developed. Pli-dCas9-sgRNA suitable for *Halomonas* sp. TD01 was designed to insert various sgRNAs, which form numerous CRISPRi repression plasmids. Multiple sgRNAs could also be inserted into pli-dCas9-sgRNA (Fig. 2a). The IPTG inducible CRISPRi system functioned well in *Halomonas* sp. TD01 for manipulating gene expression levels, achieving elongation of cell sizes, controllable PHBV copolymer monomer ratios, and enhanced PHB synthesis (Figs. 3, 4; Tables 1, 2).

FtsZ plays a crucial role in cytokinesis and assembles to form the Z rings during the cell division process [17]. Inhibition of *ftsZ* gene in *Halomonas* sp. TD01 resulted in formation of filamentous cells compared with short bar wild type of control groups (Fig. 3a). This phenomenon demonstrated the effectiveness of the established CRISPRi system for *Halomonas* sp. TD01, even though some *Halomonas* cells in the experimental groups still maintained their short bar shape possibly due to plasmid instability (Fig. 3a). Eventually, it is important to integrate the CRISPRi elements into the genome of *Halomonas* sp. TD01 to lower metabolic pressure on the bacteria, and to reduce the cost of antibiotics, as well as to improve the plasmid stability.

*Halomonas* sp. TD01 is able to produce PHBV using propionic acid as the precursor [14]. By increasing propionic acid concentration in the culture medium, *Halomonas* sp. TD01 can accumulate PHBV with a slightly improved ratio of 3HV (3% 3HV per 1 g/L propionic acid) [14]. The *prpC* gene knockout *Halomonas* sp. TD01 was very sensitive to 1 g/L propionic acid, growing slowly yet accumulated PHBV with higher 3HV content [14]. In this study, we achieved to regulate *prpC* gene at different expression levels without affecting cell growth (Table 1): all the recombinants and the controls grew well reaching approximately 14 g/L CDW that contains around 75% PHBV in the presence of 1 g/L propionic acid. Different CRISPRi inhibition targets resulted in variable repression effects (Table 1). Among the *prpC* gene inhibition targets, prpC6, prpC7 and their combined inhibition sites led to the highest improvement on 3HV ratio in PHBV copolymers (Table 1), reaching around 12–13% 3HV in the PHBV. Compared with *prpC* gene knockout approach which generates a fixed 3HV ratio in the PHBV, the CRISPRi platform provided a flexible regulation on 3HV contents in the PHBV (Table 1).

TCA cycle plays a crucial role in cell growth [22]. Genes involved in the TCA cycle are essential, as is the case of *gltA* gene, and therefore, they cannot be deleted from the genome of *Halomonas* sp. TD01. Thus, CRISPRi was employed to partially repress *gltA* expression under controlled intensities, allowing reduced consumption of acetyl-CoA for TCA cycle and diverging more acetyl-CoA to improve PHB production. Therefore, the constructed CRISPRi system can be used to regulate essential gene expression in *Halomonas* sp. TD01 for achieving multiple metabolic engineering goals.

## Conclusions

A CRISPRi system dedicated to the non-model organism *Halomonas* sp. TD01 was successfully constructed and proven feasible, as evidenced by changing cell morphology, copolymer PHBV structures and homopolymer PHB synthesis when the genes *ftsZ, prpC* or *gltA* were repressed under different intensities. Considering the promising application prospect of *Halomonas* sp. TD01 for PHA and the chemical industry, the established CRISPRi system is expected to be useful for more metabolic engineering applications.

## Methods

### Strains, plasmids and culture conditions

*Halomonas* sp. TD01 was isolated from Aydingol Lake of Xinjiang Province, China, and stored in CGMCC (China General Micro-biological Culture Collection Center, Beijing). The collection number is 4353. *E. coli* S17-1 was used as a vector donor strain in conjugation. *E. coli* S17-1 was cultured in LB-20 medium. The ingredients of LB-20 medium are (g/L): 20 NaCl, 10 tryptone, 5 yeast extract. *Halomonas* sp. TD01 and its derivative strains were all cultivated in LB-60 medium. The ingredients of LB-60 medium are (g/L): 60 NaCl, 10 tryptone, 5 yeast extract. Chloramphenicol concentration used in this study was 25 µg/mL. All the strains and plasmids used in this study are listed (Additional file 1: Table S1).

### Construction of recombinant strains

#### Plasmid construction

The plasmids used in this study are listed in Additional file 1: Table S1. Molecular cloning experiments were carried out according to manufacturers’ instructions or standard procedures. Kits for DNA purification and isolation of high quality plasmids were purchased from Qiagen (Shanghai, China). Restriction enzymes and DNA modification enzymes were provided by New England Biolabs (USA).

Based on plv-dCas9-sgRNA constructed in our previous study and pSEVA321 (kindly donated by Dr. Victor de Lorenzo of CSIC, Spain) [5, 29], a new plasmid termed pli-dCas9-sgRNA was successfully constructed, containing the dCas9 protein, restriction enzyme sites for sgRNA sequence insertion, P_trc_ promoter, RK2 origin, chloramphenicol resistance selection marker, and the origin of transfer (oriT) for conjugation (Fig. 2a). Compared with the P_tet_ promoter induction system in our previous study [5], P_trc_ promoter in plv-dCas9-sgRNA plasmid was found to be more effective as P_tet_ promoter could not function well in *Halomonas* sp. TD01. The IPTG inducible P_trc_ promoter was more sensitive for induction.

To construct pli-dCas9-sgRNA, DNA fragments containing the dCas9 and sgRNA domain were amplified from PCR using plv-dCas9-sgRNA as the template, and then inserted into pSEVA321, forming pSEVA-dCas9-sgRNA. Multiple cloning sites (MCS) including *Xma*I*, Xba*I and *Spe*I were introduced into pSEVA-dCas9-sgRNA to form pli-dCas9-sgRNA. *Xma*I and *Xba*I were introduced upstream of the sgRNA expression cassette, while *Spe*I was inserted downstream of it. *Xba*I and *Spe*I are isocaudomers that create the same cohesive end after digestion and are required for sgRNA biobrick assembly (Fig. 2). By digesting pli-dCas9-sgRNA1 vector and PCR fragment containing sgRNA2 from pli-dCas9-sgRNA2 with *Xma*I*/Xba*I and *Xma*I*/Spe*I, respectively, pli-dCas9-sgRNA1sgRNA2 plasmid was formed after ligation. In addition, *Xma*I and *Xba*I restriction sites were reconstructed for the next round of sgRNA biobrick insertion (Fig. 2). In this way, multiple sgRNA biobricks could be inserted into one vector backbone for manipulating multiple genes simultaneously.

The 20–23 bp sgRNA complementary sequence was designed via primers (Additional file 1: Table S2). The forward and reverse primers were annealed to be a double-stranded DNA fragment precisely fitting the pli-dCas9-sgRNA vector, which was cleaved by *BspQI* enzyme. The reconstructed plasmid harboring the designed sgRNA sequence was formed after ligation. This technique allowed convenient changes in the complementary region to suit any interested gene.

To construct pli-dCas9-ftsZ(1-2), pli-dCas9-prpC(1-7) and pli-dCas9-gltA(1-4), sgRNA primers were annealed by temperature gradient PCR, the PCR product was then cut by BspQI at 50 °C for 2 h and purified. The pli-dCas9-sgRNA vector was digested by BspQI at 50 °C for 4 h and purified via electrophoresis. The inhibition plasmid was formed after ligation of the vector and PCR product. To prepare electro-competent *E. coli* S17-1, 3 mL volume of overnight cell culture was collected after centrifugation, the cells were then washed twice with ice-cold 10% glycerol. The ligation product was transformed into *E. coli* S17-1 via electroporation. Cells after transformation were placed on a LB-20 plate in the presence of 25 µg/mL chloramphenicol and cultivated overnight. Positive colonies were verified by PCR. Subsequently, the constructed plasmid was transformed into *E. coli* S17-1 and then conjugated into *Halomonas* sp. TD01.

To construct pli-dCas9-prpC6prpC7, DNA fragment containing prpC6 sgRNA, *Xma*I and *Spe*I restriction sites were amplified from PCR as the insert DNA, using pli-dCas9-prpC6 as the template. The insert DNA fragment was then cut by *Xma*I and *Spe*I. Vector pli-dCas9-prpC7 was restricted using *Xma*I and *Xba*I. After ligation of the insert DNA and the vector, pli-dCas9-prpC6prpC7 was formed, containing the reconstructed *Xma*I and *Xba*I restriction sites. The plasmid was then conjugated from *E. coli* S17-1 to *Halomonas* sp. TD01.

### Designing sgRNA for repressing *prpC* gene

The sgRNAs were designed in the promoter region or near the ATG sequence in the targeted gene, and were right after a NGG sequence, namely, PAM sequence (protospacer adjacent motif sequence) [4]. All the sgRNAs could bind to the non-template DNA strand with sequence specificity. As shown in Fig. 4a, seven sgRNAs were designed along the *prpC* gene, mostly near the start site of the coding sequence or within 260 bp from the ATG sequence. In details: sgRNAs *prpC1, prpC3, prpC6, prpC7, prpC4, prpC5* and *PrpC2* were designed 15 bp downstream from ATG sequence, from position 16 to 38, after the PAM sequence CGG (from position 13 to 15), 35 bp downstream from ATG sequence, from position 36 to 58, after the PAM sequence CGG (from position 33 to 35), 88 bp downstream from ATG sequence, from position 89 to 111, after the PAM sequence CGG (from position 86 to 88), 127 bp downstream from ATG sequence, from position 128 to 150, after the PAM sequence CGG (from position 125 to 127), 190 bp downstream from ATG sequence, from position 191 to 213, after the PAM sequence CGG (from position 188 to 190), 237 bp downstream from ATG sequence, from position 238 to 260, after the PAM sequence GGG (from position 235 to 237) and 656 bp downstream from ATG sequence, from position 657 to 679, after the PAM sequence GGG (from position 654 to 656), respectively.

### Conjugation into *Halomonas* sp. TD01

Plasmids were transferred from *E. coli* S17-1 to *Halomonas* sp. TD01 through conjugation. Both the donor and recipient cells were cultured overnight. Then 10 µL *E. coli* S17-1 and 10 µL *Halomonas* sp. TD01 were mixed and placed on a LB-20 plate without antibiotics and cultured in 37 °C. After four hours, the colonies from the LB-20 plate were picked up and cultured in LB-60 plates with chloramphenicol. The ingredients of LB-20 medium are (g/L): NaCl 20, tryptone 10, yeast extract 5.

### Growth curve establishments

The cells were cultivated in LB-60 for 3 h, then 1 mM IPTG was added to induce the CRISPRi system. The total time of cell cultivation was 12 h. Then 200 µl bacterial fluid was inoculated into each well of a 96-well plate, with three parallel samples for each strain. Each sample in the well was diluted with LB-60 medium until OD600 reached 0.001. LB-60 medium was used as the blank control. All the samples was then cultured for 24 h under continuous rotary shaking (Thermo Scientific Varioskan Flash, Thermo Scientific, USA). OD600 was examined every half hour. After a deduction of the blank control, the average OD600 of each sample at each time point was calculated and used for growth curves.

### Environmental scanning electron microscope (ESEM) analysis

The cells were harvested by centrifugation at 10,000*g* for 1 min, and were first fixed with 2.5% (v/v) glutaraldehyde for more than 4 h, followed by washing with 0.1 M phosphate-buffered saline (PBS) (pH 7.3) (3 times, 10 min each). Afterwards, the fixed cells were washed by ethanol in a concentration gradient (v/v) of 50, 70, 80, 90 and 100% successively, and then dehydrated by tertiary butyl alcohol mixed with ethanol in a ratio of 1:1. The cells were treated with pure tertiary butyl alcohol and used for imaging after lyophilization. The bacteria were imaged using a environmental scanning electron microscope (FEI Quanta 200, America) and analyzed utilizing XT Microscope Server imaging software.

### Shake flask experiments in PHA production

After the plasmid was conjugated into *Halomonas* sp. TD01, the positive colonies were PCR verified. Then we obtained experimental TD strains, TD-ftsZ(1-2), TD-prpC(1-7), TD-prpC6prpC7 and TD-gltA(1-4), with each harboring a CRISPRi inhibition plasmid. Wild type *Halomonas* sp. TD01 and TD-sgRNA were used as the controls.

For production of PHA in *Halomonas* sp. TD01 and its derivate strains, the bacteria were cultivated in mineral medium (MM) containing (g/L): 60 NaCl, 30 glucose, 1 yeast extract, 1 NH_4_Cl, 0.2 MgSO_4_, 9.65 Na_2_HPO_4_-12H_2_O, 1.5 KH_2_PO_4_, 10 mL/L trace element solution I and 1 mL/L trace element solution II. Glucose concentration of the MM medium culturing *Halomonas* sp. TD01, TD-sgRNA, TD-ftsZ1 and TD-ftsZ2 in feasibility study of the constructed CRISPRi for *Halomonas* sp. TD01 was 20 g/L. The composition of trace element solution I was (g/L): 5 Fe(III)-NH_4_-citrate, 2 CaCl_2_, 1 M HCl. The trace element solution II contains (mg/L): 100 ZnSO_4_-7H_2_O, 30 MnCl_2_-4H_2_O, 300 H_3_BO_3_, 200 CoCl_2_-6H_2_O, 10 CuSO_4_-5H_2_O, 20 NiCl_2_-6H_2_O, 30 NaMoO_4_-2H_2_O, 1 M HCl at pH 9.0 [13].

For shake flask experiments, seed cultures were grown in 37 °C in LB-60 medium for 12 h at 200 rpm on a rotary shaker (HZQ-F160, HDL, Harbin, China). For each experimental group, three parallel samples were set. For each shake flask, 3 mL volume of the seed culture was inoculated in MM medium with 25 µg/mL chloramphenicol. The total volume of the shake flask was 50 mL. After 12 h of cultivation, IPTG was added to a final concentration at 1 mM to induce the CRISPRi inhibition system. Exceptionally, for the feasibility study of CRISPRi in *Halomonas* sp. TD01, TD-sgRNA, TD-ftsZ1 and TD-ftsZ2, the bacteria were cultured for 8 h before induction. The total time of cell culture was 48 h.

The bacteria were harvested, centrifuged at 10,000×*g* and washed once with distilled water. The cells were lyophilized and CDWs were measured. After methanolysis in chloroform at 100 °C for 4 h and cooling to room temperature, 1 mL deionized water was added. The components of the samples were then mixed by vortexing. Stratification then appeared in the sample solution, with the organic phase containing PHA. The samples were stood still for 1.5 h, and 1 mL chloroform containing PHA from the bottom layer was taken by syringe for gas chromatograph analysis. The samples were then analyzed by a gas chromatograph (GC-2014, SHIMADZU, Japan) and GCsolution software was employed to determine the PHA content [5]. Analytically pure PHB and PHBV copolymer (Sigma-Aldrich) were used as the standard samples to investigate 3HB and 3HV monomer quantities, respectively.

### Real-time PCR

All the TD strains were cultivated in LB-60 medium for 3 h, then 1 mM IPTG was added to induce the CRISPRi system. The total time of cell cultivation was 12 h. The total RNA was isolated from *Halomonas* sp. TD01 and recombinant *Halomonas* sp. TD01 strains by using the RNA prep pure Cell/Bacteria Kit (Tiangen, Beijing, China). The Fastquant RT Kit (Tiangen, Beijing, China) was used to synthesize the cDNA for mRNA analysis. 16S rRNA was used as the inner standard, real-time PCR (RT-PCR) was carried out for mRNA analysis with SuperReal PreMix (SYBR Green) (Tiangen, Beijing, China).

The total extracted RNA concentration was measured by Nanodrop 2000 spectrophotometer (Thermo Fisher Scientific, USA) to design a concentration gradient for cDNA synthesis (using random primers following standard procedures described in the manufacturer’s product specification). The cDNA was used immediately in the RT-PCR analysis. The linear interval of total RNA was analyzed as a standard for the following experiments to adjust the quantity of the template within its linear range, so that the fluorescence quantitative results could be designed within a rational range. All samples were prepared with three parallel groups to obtain results of ΔCt values from the outputs of RT-PCR.

## Additional file

**Additional file 1: Table S1.** Strains and plasmids used in this study. **Table S2.** Primers used for genetic manipulations. **Fig. S1.** Growth of recombinant *Halomonas* sp. TD01 harboring CRISPRi system targeting *ftsZ* gene in LB-60 medium. **Fig. S2.** Growth of recombinant *Halomonas* sp. TD01 with controllable *prpC* gene transcription via CRISPRi in LB-60 medium. **Fig. S3.** Growth of recombinant *Halomonas* sp. TD01 harboring CRISPRi system targeting *gltA* gene in LB-60 medium. **Fig. S4.** RT-PCR tests of *gltA* transcription levels in recombinant *Halomonas* sp. TD01.
