# Supplementary material for: Engineering Halomonas species TD01 for enhanced polyhydroxyalkanoates synthesis via CRISPRi
Source: Microb Cell Fact. 2017 Apr 6;16:48. doi: 10.1186/s12934-017-0655-3 (PMC5382479; doi:10.1186/s12934-017-0655-3)
Supplement: Supplementary file 1 — Additional file 1: Table S1. Strains and plasmids used in this study. Table S2. Primers used for genetic manipulations. Fig. S1. Growth of recombinant Halomonas sp. TD01 harboring CRISPRi system targeting ftsZ gene in LB-60 medium. Fig. S2. Growth of recombinant Halomonas sp. TD01 with controllable prpC gene transcription via CRISPRi in LB-60 medium. Fig. S3. Growth of recombinant Halomonas sp. TD01 harboring CRISPRi system targeting gltA gene in LB-60 medium. Fig. S4. RT-PCR tests of gltA transcription levels in recombinant Halomonas sp. TD01. [file 12934_2017_655_MOESM1_ESM.docx]

**Engineering *Halomonas* species TD01 for Enhanced Polyhydroxyalkanoates Synthesis via CRISPRi**

Wei Tao, Li Lv, Guo-Qiang Chen

School of Life Sciences, Tsinghua University, Beijing 100084, China

Table S1 Strains and plasmids used in this study

| Strains/plasmids | Description | Reference/source |
| --- | --- | --- |
| TD01 | Wild type *Halomonas* sp. TD | (Tan et al., 2011) |
| *E. coli* S17-1 | recA,thi-1.pro,hsdR, RP4-2-Tc::Mu-Km::Tn7 | This study |
| plv-dCas9-gRNA | sgRNA element inserted into plv-dCas9 (mcs) plasmid, Cm^R^ | (Lv et al., 2015) |
| pSEVA-dCas9-sgRNA | dCas9 and sgRNA element inserted into pSEVA321 plasmid, Cm^R^ | This study |
| pli-dCas9-sgRNA | Multiple cloning sites (MCS) inserted into  pSEVA-dCas9-sgRNA, Cm^R^ | This study |
| pli-dCas9-ftsZ(1-2) | pli-dCas9-sgRNA plasmid targeting *ftsZ*, Cm^R^ | This study |
| pli-dCas9-prpC(1-7) | pli-dCas9-sgRNA plasmid targeting *prpC*, Cm^R^ | This study |
| pli-dCas9-prpC6prpC7 | pli-dCas9-sgRNA plasmid targeting *prpC*, Cm^R^ | This study |
| pli-dCas9-gltA(1-4) | pli-dCas9-sgRNA plasmid targeting *gltA*, Cm^R^ | This study |

Chloramphenicol concentration used in this study was 25 µg/ml.

Table S2 Primers used for genetic manipulations

| Primers | Sequence (5’-3’) |
| --- | --- |
| ftsZ1-5' | AAACCCGTCTCCTGAACCGCGCTTG |
| ftsZ1-3' | AACCAAGCGCGGTTCAGGAGACGGG |
| ftsZ2-5' | AAACTTCGATATTACTTTCGACCATG |
| ftsZ2-3' | AACCATGGTCGAAAGTAATATCGAAG |
| prpC1-5' | AAACCACGGAGTCCTGCGCTGTTTTG |
| prpC1-3' | AACCAAAACAGCGCAGGACTCCGTGG |
| prpC2-5' | AAACTTCATTAGCACCGCCGTGCAGC |
| prpC2-3' | AACGCTGCACGGCGGTGCTAATGAAG |
| prpC3-5' | AAACTGTGGTACCAGCGCTTTGTCCA |
| prpC3-3' | AACTGGACAAAGCGCTGGTACCACAG |
| prpC4-5' | AAACTTGGTGATGTAGCCATCAAGCT |
| prpC4-3' | AACAGCTTGATGGCTACATCACCAAG |
| prpC5-5' | AAATCCAGCACGGATTTCAGGGCATC |
| prpC5-3' | AACGATGCCCTGAAATCCGTGCTGGA |
| prpC6-5' | AAAATCAAAACCACGGTAGGTTAATC |
| prpC6-3' | AACGATTAACCTACCGTGGTTTTGAT |
| prpC7-5' | AAAGACTTCTTCAAACTTTGCCTTCT |
| prpC7-3' | AACAGAAGGCAAAGTTTGAAGAAGTC |
| gltA1-5' | AAAGCCTCCTTTCGAATTCGGTTT |
| gltA1-3' | AACAAACCGAATTCGAAAGGAGGC |
| gltA2-5' | AAACCAAGTGTGCCGGAATACAT |
| gltA2-3' | AACATGTATTCCGGCACACTTGG |
| gltA3-5' | AAACGCGTACGTCGATGACGTCG |
| gltA3-3' | AACCGACGTCATCGACGTACGCG |
| gltA4-5' | AAAGGAGAGGGGGCCATGAAGCC |
| gltA4-3' | AACGGCTTCATGGCCCCCTCTCC |

All oligonucleotides were synthesized by Invitrogen (Life Technology, USA)

Fig. S1

Fig. S1 Growth of recombinant *Halomonas* sp. TD01 harboring CRISPRi system targeting *ftsZ* gene in LB-60 medium. Data shown were the average of three parallel experiments.

TD01, *Halomonas* sp. TD wild type; TD-sgRNA, TD01 strain harboring the pli-dCas9-sgRNA plasmid without any target site; TD-ftsZ1, TD-ftsZ2, TD01 strains harboring the CRISPRi plasmids pli-dCas9-ftsZ1 and pli-dCas9-ftsZ2*,* respectively.

Fig. S2

Fig. S2 Growth of recombinant *Halomonas* sp. TD01 with controllable *prpC* gene transcription via CRISPRi in LB-60 medium. Data shown were the average of three parallel experiments.

TD01, *Halomonas* sp. TD wild type; TD-sgRNA, TD01 strain harboring the pli-dCas9-sgRNA plasmid without any target site; TD-prpC(1-7), TD-prpC6prpC7, TD01 strains harboring the pli-dCas9-sgRNA plasmid with different sgRNA targets of gene *prpC*.

Fig. S3

Fig. S3 Growth of recombinant *Halomonas* sp. TD01 harboring CRISPRi system targeting *gltA* gene in LB-60 medium. Data shown were the average of three parallel experiments.

TD01, *Halomonas* sp. TD wild type; TD-sgRNA, TD01 strain harboring the pli-dCas9-sgRNA plasmid without any target site; TD-gltA(1-4), TD01 strains harboring the pli-dCas9-sgRNA plasmid with different sgRNA targets of gene *gltA*.

Fig. S4

Fig. S4 RT-PCR of *gltA* transcription levels in recombinant *Halomonas* sp. TD01.

All data were the average of three independent studies with standard deviations. Mean±S.E. (n=3). * and ** indicate p<0.05 and p<0.01, respectively.

TD01, *Halomonas* sp. TD wild type; TD-sgRNA, TD01 strain harboring the CRISPRi system without any target site; TD-gltA(1-4), TD01 strains harboring the CRISPRi system with different sgRNAs targeting *gltA* gene.
